# Development and psychometric testing of the ‘barriers to physical activity during pregnancy scale’ (BPAPS)

**DOI:** 10.1186/s12889-021-11511-3

**Published:** 2021-07-29

**Authors:** Leila Amiri-Farahani, Katayon Ahmadi, Syedeh Batool Hasanpoor-Azghady, Sally Pezaro

**Affiliations:** 1 Department of Reproductive Health and Midwifery, Nursing Care Research Center, School of Nursing and Midwifery, Iran University of Medical Sciences, Postal code, Tehran, 1996713883 Iran; 2 School of Nursing and Midwifery, Iran University of Medical Sciences, Tehran, Iran; 3 Faculty of Health and Life Sciences, Coventry University, Coventry, UK

**Keywords:** Barriers, Physical activity, Exercise, Pregnancy, Validated scale

## Abstract

**Background:**

Pregnancy can affect the amount of physical activity that women engage in, so ensuring adequate physical activity in pregnant women can be a challenge. Therefore, there is a need to explore and identify barriers to physical activity in this population. Consequently, this study was conducted in order to develop and validate a scale to assess barriers to physical activity in pregnant populations.

**Methods:**

The study was conducted in two phases. During phase 1, a comprehensive review of the most relevant literature from electronic databases on barriers to physical activity was carried out and appropriate scale items were extracted using a deductive approach. During phase 2, the psychometric properties of the extracted scale items were determined using face, content and construct validity, internal consistency and stability. Qualitative and quantitative face validity was assessed via face-to-face interviews with 30 and 10 pregnant women, respectively. To confirm the qualitative and quantitative content validity, 10 experts in the field of research and instrument design were invited to complete the resulting scale. To assess construct validity, the scale items were further tested among 320 pregnant women attending perinatal clinics at health centers in Ilam, Iran, where data were collected via continuous sampling. The internal consistency and stability of the study were measured by Cronbach’s alpha and intra-class correlation coefficient (ICC), respectively.

**Results:**

Following a review of the relevant literature, 48 items for the BPAPS were extracted. Subsequent to the assessment of face and content validity, the number of items was reduced to 38. Through a subsequent exploratory factor analysis, the number of items dropped further to 29. These items were then structured under four major factors. Finally, the internal consistency and stability of the scale was confirmed by a Cronbach alpha coefficient of 0.824 and a test-retest reliability score of 0.87.

**Conclusion:**

Findings show that the 29-item scale to assess barriers to physical activity in pregnant populations is a valid and appropriate instrument.

**Supplementary Information:**

The online version contains supplementary material available at 10.1186/s12889-021-11511-3.

## Background

Pregnancy is a life-changing event for women, and may significantly affect the amount of physical activity they engage in. Yet meeting the guidelines for physical activity during pregnancy can be challenging [1], despite the numerous physical and mental health benefits are associated with it [2]. Current guidance recommends regular participation in a wide range of moderate-intensity aerobic and recreational activities during an uncomplicated pregnancy [3]. Nevertheless, some pregnant women engage in such activities less frequently [4]. This is concerning, as engaging in the recommended amount of moderate-intensity physical activity during pregnancy can significantly reduce the risk of gestational diabetes, preterm delivery and preeclampsia [2].

Additional health benefits of physical activity also include healthy postpartum weight loss, higher scores on psychosocial health measures [5], improved sleep [6], and reduced postpartum depression [7]. Considering these outcome effects and the effect of physical activity during pregnancy upon birth [8], it would be advantageous for every pregnant woman to engage in some form of physical activity. Accordingly, there is a need to assess the barriers to physical activity in pregnant populations.

Various qualitative and quantitative studies have identified a number of barriers to physical activity during pregnancy [9–13]. Reported intrapersonal barriers include pregnancy symptoms, family and child-rearing responsibilities and activities, lack of personal motivation, time and job requirements, perceptions of sufficient daily physical activity, fear of fetal injury, and lack of physical activity habits [10, 14]. Additional interpersonal barriers include lack of social support, overprotective family members, receiving conflicting advice from others, social isolation, lack of knowledge of physical activity [4], and the lack of an exercise companion [14]. Factors such as cost-related concerns related to physical activity, lack of means of transport, and weather-related concerns have also been identified as environmental barriers [15]. In response, it has been suggested that multilevel interventions targeting individuals, social environments, physical environments, and policies are now required to achieve population change in physical activity [16].

As well as assessing barriers, Harrison et al. (2018) summarized a number of quantitative and qualitative studies assessing attitudes and enablers of physical activity in pregnant women. Yet, in relation to socio-ecological findings, some barriers were based solely on the results of qualitative studies, and therefore the generalizability of such findings to other populations is ambiguous [12, 13]. As such, these tools cannot be wholly considered valid and reliable. Additionally, the results of an alternate review have confirmed that some barriers such as pregnancy discomfort, women’s fear of exercise during pregnancy, and uncertainty about the safety of exercise during pregnancy have not yet been considered in the development of assessment tools to identify barriers to physical activity/exercise [13]. As such, a comprehensive scale which can measure all aspects in relation to the barriers to physical activity during pregnancy is now required.

As yet, there is only one other scale developed to identify barriers to physical activity. This EBB: Exercise Benefits/Barriers Scale is considered to be a general tool to be used for whole populations [12, 13, 17]. Yet since this scale also does not measure all aspects of barriers to physical activity during pregnancy, the present study was conducted to develop and psychometrically test the ‘Barriers to Physical Activity during Pregnancy Scale’ (BPAPS). As there are multiple levels of influence on physical activity, and active living is associated with different social and environmental variables [16], this scale takes a socio-ecological approach and is intended to provide deeper quantitative insights in relation to the barriers to pregnant women engaging in physical activity.

## Methods

The current methodological study consists of the following two consecutive phases:

### Phase 1: item generation and scale development

During phase one, a comprehensive review of the most relevant literature from electronic databases was carried out to identify scale items relating to the barriers to physical activity in pregnant women. Data were extracted from quantitative, qualitative and mixed method studies, along with both systematic and non-systematic literature reviews. To identify potentially relevant studies, PubMed/Medline and Web of Science electronic databases were systematically searched. All articles published in during the last 20 years, using the referencing period between 1997 and 2017 were included. A comprehensive search strategy was developed, combining the following keywords: [(barriers OR constraints OR perceptions OR attitudes) AND (physical activity OR exercise OR motor activity) AND (pregnancy OR pregnant women OR antenatal OR prenatal)]. Additional relevant studies were identified by manually searching the reference lists of included studies and by citation tracking. In addition, experts in the field were contacted to identify potentially relevant studies. Studies were included if they had reported any perceived barriers to physical activity among pregnant women as either their primary or secondary outcomes.

Among the articles included, a number of reviews, along with both qualitative and quantitative studies presented findings in relation to unvalidated tools [12, 13]. Such findings were only used to inform items included within the early BPAPS if they related directly to social and environmental variables which influenced physical activity in order to support our socio-ecological approach.

### Phase 2: validity and reliability of the scale

In order to determine the psychometric properties of the BPAPS at this stage, the face, content, and construct validity as well as the reliability were assessed.

#### Face validity

The present study used continuous sampling. Pregnant women who had been referred to a perinatal clinic within the health centers of Ilam were invited to participate if they were of Iranian nationality, aged between 16 and 45 years, between 10 and 37 weeks pregnant and able to safely engage in physical activity during pregnancy. Clinics providing perinatal services in Ilam were first divided into two strata: health centers and health bases. Subsequently, the principle investigator selected 8 out of 10 health centers and 3 out of 5 health bases at random. Then, via the proportional allocation method, the sample size within each health center or health base was set equal to be proportional to the number of pregnant women assigned to each setting.

Face validity of the BPAPS was assessed using both qualitative and quantitative methods. Firstly, face-to-face interviews with 30 pregnant women were conducted to determine qualitative face validity in which factors such as determining the level of difficulty in understanding words and phrases, their relationship with other dimensions of the scale, as well as the possibility of ambiguity or misinterpretation of certain items were thoroughly assessed. During these interviews, participants were invited to complete the BPAPS and specifically comment upon the clarity and comprehensibility of each item and its phrasings. They were also asked to comment upon any difficulties in reading each item and on any interrelationships between them. Lastly, participants were asked about how well the scale matched their own experiences of physical activity during pregnancy, and were invited to delete or add any potential new items accordingly. Items were then refined in response to participant suggestions.

In order to determine quantitative measures for the assessment of face validity, a sub-sample of 10 participants were chosen at random and asked to rate each item, first on the basis of its importance and then, on the basis of their assessment, score each item from 1 to 5. Scores were assigned the following values: ‘absolutely essential’ (score 5), ‘essential’ (score 4), ‘moderately important’ (score 3), ‘slightly important’ (score 2), and ‘not important at all’ (score 1). In order to measure the impact score of each item, the frequency of participants, paired with item scores of 4 or 5 were multiplied by the mean importance score. In line with other studies [18, 19], if the impact score for an item was greater than 1.5, then the item was then considered appropriate and retained for further analysis. Finally, items were edited and refined further with a well-versed research expert, who was also experienced in the translation and modification of such texts.

#### Content validity

In order to confirm the content validity qualitatively, 10 experts in the field of research (faculty member of reproductive health and midwifery in Tehran and Iran University of Medical Sciences) and instrument design were selected and asked to complete the scale. After a comprehensive review of the tool and in line with best practice [20], this expert panel was asked to complete the scale and submit their detailed corrections in written form with regard to the correct use of grammar, the proper use, and the importance of the words, and the appropriateness of item allocation. Items were then refined in response to the expert panel’s suggestions.

The content validity ratio (CVR) and the content validity index (CVI) were used to determine the quantitative validity of the content. To determine the CVR, experts were asked to rate the necessity of each item using a three-point Likert scale (1 = item is not essential, 2 = item is useful but not essential, 3 = item is essential). Based on the feedback of these experts, the CVR value was measured collectively. Expert responses were quantified and the content validity ratio was determined. Scores relating to the ‘essential’ option [3] were given a score of 1 and the other two options were given a score of zero. Then, according to the following formula, the content validity ratio was calculated. The score obtained was calculated with the table provided by Lavache in terms of acceptability [21]. In this formula, Ne represents the number of people who selected the ‘essential’ option and N represents the number of specialist respondents.

$$ CVR=\frac{\left({n}_e-\frac{N}{2}\right)}{N\div 2} $$

According to Lawshe′s table (minimum values of CVR), when the number of expert responses reaches 10, items with CVR value of 0.62 or higher are considered appropriate [21].

The content validity index (CVI) for each item was then calculated using the Waltz and Bussel criteria [22]. Experts were asked to rate the relevance of the items on a four-point Likert scale accordingly (1 –irrelevant, 2 –somewhat relevant, 3–acceptably relevant, and 4–completely relevant). For the calculation of the CVI of each item (I-CVI), the number of experts who gave the scores of 3 or 4 per item were divided by the total number of expert participants [23]. In line with previous studies [24], if the item’s calculated CVI was greater than 0.79, it was considered appropriate. However, if the calculated CVI fell between 0.70 and 0.79, the items were modified, and if an item’s calculation was found to be less than 0.7, it was removed from the scale altogether [24]. Finally, the average of all I-CVIs for items was used to calculate the scale-level using the CVI / average calculation method (S-CVI / Ave) [24].

#### Assessment of construct validity

The construct validity of the BPAPS was assessed using exploratory factor analysis and known-groups comparison. Factor analysis evaluates the interrelationships between the items and classifies the interrelated them [25, 26]. The minimum sample size for factor analysis is equal to the number of items multiplied by 5 to 10 [27]. Bujang et al. (2013) states that the required number of responders for EFA is between 3 and 10 persons per item, or a total of 100 to 200 responders [28]. Our sample size was 320 women. This is 8 times higher than the remaining items at the end of the previous psychometric assessment. In the present study, continuous sampling was performed from October 2018 to January 2019. Scales were filled out on the basis of self-reported inventories. Following completion, participant data was stored securely and transferred to an SPSS data file.

For item analysis, the inter-item correlation and the correlation between each individual item was used to evaluate the overall BPAPS score. If the correlation coefficient score of at least one item or the overall BPAPS score was less than 0.3, the item was removed from the scale [27]. Also, if the correlation coefficient between the two items was > 0.7, one item was removed from the scale [29].

The main component factor analysis was performed using an equamax rotation. In order to determine the appropriateness of the factor analysis model, the sampling adequacy and the number of factors, Bartlett’s test of sphericity, Kaiser-Meyer-Olkin (KMO) test, and scree plot of eigenvalues were used. A minimum factor loading of 0.3 was employed to maintain the items in the extracted factors.

The known-group comparison method was used to determine the validity of the construct [25, 26]. This technique helps to identify the ability of the intended scale to separate groups with divergent experiences [30]. In the present study, the known groups consisted of women with and without a history of miscarriage. This was because women with a history of miscarriage can perceive more barriers and fear in relation to engagement in physical activity during subsequent pregnancies [31, 32]. Here, the physical activity barriers in both groups were measured using the BPAPS. Scores were compared using an independent sample t-test. Subsequently, in order to assess discriminatory validity, correlations between factors within our exploratory factor analysis were explored.

#### Reliability assessment

The reliability of the BPAPS was assessed using internal consistency and stability assessment techniques. Cronbach’s Coefficient alpha was used as an internal consistency assessment measure. Cronbach’s alpha > 0.7 reflects a satisfactory internal consistency [33]. Stability assessment over time was performed using the test-retest reliability method. Participants completed the BPAPS twice, with a two-week interval between sessions. The intraclass correlation coefficient (ICC) was used to assess the relationship between test-retest scores. ICC scores of 0.8 or higher denote satisfactory stability [34].

### Statistical analysis

Analysis was done using SPSS version 21.0 statistical software and consisted of the following: 1) Calculating the distribution of data. Here, the skewness, and the kurtosis of each variable showed the normality of the variables for the application of parametric analysis; 2) Descriptive statistics including frequency distribution, central tendency and index of dispersion including mean and standard deviation to describe the characteristics of the participants in the study; 3) Use of equamax rotation for the factor analysis to refine the items; 4) Use of independent sample t-test for comparison between women with and without history of miscarriage; 5) Calculating Cronbach’s alpha to examine internal consistency; and 6) Calculating the ICC between the test scores and the retest scores.

### Ethical considerations

The study was approved by the Ethical Committee of the Research Council of Iran University of Medical Sciences (Number: IR.IUMS.REC.1397.1143), Tehran, Iran. Study participants were personally informed about the aims and importance of the study. They were assured of the anonymity and confidentiality of their information and were free to participate or withdraw from the study at any time, without reason.

## Results

### Phase 1

Following our comprehensive review of the literature, a total of 63 articles were obtained, of which 35 articles met our inclusion criteria. Of these, 5 were reviews of the literature (2 non systematic and 3 systematic), 17 reported quantitative findings and 13 reported qualitative findings. From these 35 articles, 240 potentially relevant items were extracted by the reseach team, as articles were read and re-read separately. The items were extracted from different studies reported in supplementary Table S1. Duplicate items (*n* = 174) were removed. The research team then worked together to examine the remaining 66 items collectively. Similar items were removed, while overlapping items were merged via a succession of refinements through a series of academic discussions. The final consensus number of items pooled to enter the reliability and validity phase of this study was 48 (supplementary Table S2).

### Phase 2

#### Face validity

With regard to the qualitative assessment of face validity, text-based items were refined following a series of academic discussions, and then again by participants. Following the quantitative assessment of face validity, two items with an impact score of less than 1.5 were subsequently deleted (supplementary Table S3).

#### Content validity

With regards to the qualitative assessment of content validity, 12 items were again refined. The CVR and CVI were then calculated to determine which items would be included in the finalized BPAPS. Based on the CVR, the minimum acceptable value of CVR for 10 experts is 0.62; six items with a CVR value of less than 0.62 were subsequently removed from the scale. In addition, two items with an item-level CVI (I-CVI) below 0.7 were removed from the scale. Three items with an I-CVI of 0.75–0.79 were revised. The revised items received an I-CVI of 0.815, 0.732, 0.734 and 0.722, respectively. The scale-level CVI (S-CVI) of the whole BPAPS was measured at 0.824. According to Polit and Beck, the S-CVI / Ave of 0.9 or higher reflect excellent content validity [23]. The total items were obtained after face and content validity before doing construct validity was reported in supplementary Table S4.

#### Construct validity

The construct validity of the BPAPS was assessed using exploratory factor analysis and known-group comparison. The key component of the exploratory factor analysis was performed on the 38-item BPAPS in the exploratory factor analysis. The KMO test result was 0.822, suggesting adequacy of sampling. In addition, the Bartlett’s test showed a strong relationship between the items (*P* value < 0.001) suggesting that the factor analysis model was suitable. Factors with an eigenvalue greater than one were extracted. Scree plot revealed a four-factor scale structure. The suppressed point 0.3 was considered to be the minimum load factor for holding the items in the extracted factors. The extracted factors explained 53.911% of the total variance. Following equamax rotation, factors 1 to 4 explained 26.206, 10.043%, 9.274% and 8.388% of the variance, respectively. Items were then assigned to the factors with the highest load factor.

Overall consensus was reached by the research team via a succession of academic discussions. Any differences in opinion were resolved in this same way. Ultimately, nine items with an operating load below 0.3 were removed. Finally, 29 items were divided into four factors: first factor = 10 items, second factor = 5 items, third factor = 5 items, and fourth factor = 9 items.

The characteristics of the participants and the four-factor structure of the BPAPS are shown in Tables 1 and 2. Subsequently, the scale was applied to two known groups of women with and without a history of miscarriage in order to determine the discriminatory power of the BPAPS. Current study participants were divided into two categories, both with and without history of miscarriage. The independent sample t-test showed a significant difference in scores relating to physical activity barriers. Particularly in relation to intrapersonal barriers related to pregnancy (t = − 3.82, *P* < 0.001)**,** intrapersonal barriers non-related to pregnancy (t = − 13.25, P < 0.001) subscales and in the total score between the two groups (t = − 3.44, *P* = 0.001). Yet there were no significant differences between these two groups in relation to interpersonal barrier scores (t = 2.01, *P* = 0.055) or environmental barrier scores (t = 1.44, *P* = 0.151). Correlations of the four variables are shown in Table 3; none of the correlations were above 0.7, thus confirming discriminant validity.

**Table 1:** Characteristics of the participants in the Study

Variables	Frequency (%)
Women age (y), X ± SD	27.52 ± 5.28
Education	
High school	21 (7)
Diploma	98 (32.66)
University	181 (60.33)
Occupation	
Employed	41 (13.66)
Housekeeper	259 (86.33)
Economic status	
Unfavorable	34 (11.33)
Fairly favorable	150 (50)
Favorable	116 (38.66)
History of miscarriage	
Yes	244 (81.33)
No	56 (18.66)
Number of children	
0	153 (51)
1	112 (37.33)
2	35 (11.66)
BMI before pregnancy (kg/m^2^), X ± SD	4.28 ± 26.03
Gestational age (wk), X ± SD	
10–14	67 (22.33)
15–28	123 (41)
29–42	110 (36.66)

**Table 2:** The Four-factor Structure of BPAPS

Items	Factor 1	Factor 2	Factor 3	Factor 4
I cannot be physically active because of drowsiness.	0.623		0.366	
I cannot be physically active because of lethargy/lack of energy.	0.626		0.365	
I cannot be physically active because I do not have physical activity habits.	0.84			
Pregnancy is a time for rest.	0.83			
I cannot be physically active because of the heavy feeling of pregnancy (swelling and/or weight).	0.580		0.358	
I cannot be physically active because of my abdominal size and appearance.	0.459		0.315	
I cannot be physically active because of pain (such as back pain, hip pain, and/or headache).	0.806			
I cannot be physically active because of shortness of breath.	0.656			
I am concerned by possible pregnancy complications such as miscarriages and premature labor.	0.570		0.417	
I cannot be physically active because of pregnancy gastrointestinal problems (such as nausea, vomiting, and heart burn).	0.627		0.313	
Physical activity is too hard work for me.		0.622		0.461
I do not do physical activity because of a lack of confidence in my physical ability.		0.657		
I do not have the patience to do physical activity.		0.799		
I cannot be physically active because I do not have a regular schedule in life.		0.49	0.39	
Because of family and childrearing responsibilities/activities I do not have enough time to do physical activity.		0.59		
In our society, it is not customary for pregnant women to do physical activity.			0.690	
I do not do physical activity because I do not have access to complete information about physical activity during pregnancy.			0.495	
My friends and relatives forbid me from doing physical activity during pregnancy.			0.566	0.311
The physician/midwife does not provide advice on the benefits of physical activity during pregnancy.			0.866	
The physician/midwife does not provide advice on how to do physical activity safely during pregnancy.			0.860	
Air pollution prevents me from doing physical activity outdoors.				0.429
I do not do physical activity because I do not have access to a suitable vehicle for transportation.				0.726
It is difficult for me to do physical activity in unfavorable weather (too cold/hot).			0.351	0.470
I am not able to pay for physical activities.				0.512
There are no specific physical activity programs designed for pregnant women.				0.627
Parks are unsafe and unsuitable for pregnant women to do physical activity.				0.631
I do not do physical activity because of a lack of space at home.			0.454	0.551
There is too great a distance from my home to facilities designed for physical activity.				0.680
There are very few places for me to do physical activity.				0.749
Eigenvalue	7.6	2.912	2.689	1.448
Explained variance (%)	26.206	10.043	9.274	8.388
Cumulative variance (%)	26.206	36.249	45.523	53.911

^a^ Factor 1: Intrapersonal barriers related to pregnancy, Factor 2: Non-pregnancy intrapersonal barriers, Factor 3: Interpersonal barriers, Factor 4: Environmental barriers

**Table 3:** Discriminating Validity of the Four Dimensions of Physical Activity Barriers During Pregnancy Scale

Factors of BPAPS	1	2	3	4
Intrapersonal barriers related to pregnancy	–	0.69	0.536	0.508
Intrapersonal barriers non-related to pregnancy	0.69	–	0.476	0.443
Interpersonal barriers	0.536	0.476	–	0.652
Environmental barriers	0.508	0.443	0.652	–

#### Reliability assessment

The Cronbach alpha for the 29-item BPAPS was determined to be 0.824 (Table 4) and the ICC was determined to be 0.87 (P- = 0.001) between the test and the retest. All of these results confirm the high reliability of the BPAPS. Definitively, the 29 items which remained in the final version of the BPAPS were scored on a Likert 5-point scale as follows; 5 = strong agreement, 4 = agreement, 3 = neutral, 2 = disagreement, and 1 = strong disagreement. Based on the results obtained, the total score of BPAPS ranged from 29 to 145 with a higher score associated with greater barriers to physical activity during pregnancy. The Persian and English language versions of the BPAPS were shown in supplementary Tables S5 and S6 respectively.

**Table 4:** The Cronbach’s Alpha Values for the BPAPS and its factors

Factors	Subscale	Number of Items	Internal Consistency
1	Intrapersonal barriers related to pregnancy	10	0.815
2	Intrapersonal barriers non-related to pregnancy	5	0.732
3	Interpersonal barriers	5	0.734
4	Environmental barriers	9	0.722
Total	BPAPS	29	0.824

## Discussion

The aim of this study was to develop and validate the BPAPS used to identify barriers to physical activity among pregnant women. The final version of the BPAPS was developed with a socio-ecological approach in mind, as there are multiple levels of influence on physical activity, and active living is associated with different social and environmental variables [16]. The final BPAPS included 29 items, structured under four factors, including pregnancy-related intrapersonal barriers, non-pregnancy related intrapersonal barriers, interpersonal barriers, and environmental barriers. The results showed that BPAPS has appropriate validity and reliability.

The scale’s first, second, third and fourth factors comprised 10, 5, 5 and 9 items, respectively. Items within the BPAPS may be scored as follows on a five-point Likert scale: 5 = strongly agree, 4 = agree, 3 = neutral, 2 = disagree and 1 = strongly disagree. Accordingly, the BPAPS’s total score ranges from 29 to 145 and higher scores indicate a greater barrier to physical activity.

The EBBS, designed prior to the BPAPS by Sechrist et al. [17] consisted of 42 items for the general measurement of exercise benefits/barriers. Of these 42 items, 14 were used to measure barriers and were thus divided into four factors relating to exercise milieu, time expenditure, physical exertion, and family discouragement. Although the structure of the two instrument’s factors are different, the re-examination of the EBBS barriers section showed that 6, 4, and 4 items were similarly linked to intrapersonal, interpersonal, and environmental barriers, respectively [17]. The largest number of items in each tool therefore comprised individual barriers between the two matching tools. The highest number of items for each tool contains intrapersonal barriers, suggesting some continuity between the two instruments. Yet the increased number of individual barriers in pregnancy could be usefully recognized through the use of the new and more bespoke BPAPS.

With regard to the BPAPS′s reliability, the Cronbach’s alpha coefficients of the total scale and subscales of pregnancy-related intrapersonal barriers, non-pregnancy related intrapersonal barriers, interpersonal barriers, and environmental barriers were 0.824, 0.815, 0.732, 0.734 and 0.722, respectively. The test-retest method for assessing time stability was implemented twice within a two-week period to a random sample of pregnant women, where the BPAPS’s ICC was measured as 0.87 (P- = 0.001). Similarly, the original version of EBBS has acceptable validity and reliability, as the Cronbach’s alpha coefficients for the total scale, benefits and barriers subscales were 0.95, 0.95 and 0.86, respectively [17]. Yet whilst both data collection tools appear equally reliable, the more bespoke BPAPS based upon the more relevant literature in childbearing may be more suited for use in maternity services among childbearing populations. For this reason, the BPAPS could usefully be distributed for wider use among maternity staff and facilitators of physical activity.

In another study, Duncombe et al. [35] employed a bespoke scale exploring the barriers to physical activity during pregnancy. This included 7 items including fatigue, being too busy, disliking the exercise, feeling sick, being uncertain about the safety of the exercise, being unsure which exercise is safe, and feeling uncomfortable doing the exercise during pregnancy, yet the validity and reliability of this scale have not been confirmed. Other studies have also been conducted in relation to the measurement of barriers to physical activity during pregnancy. These are largely based on intrapersonal, interpersonal, and environmental factors [4], or the possible reasons for physical inactivity during pregnancy [14, 36]. Yet since no valid and/or reliable tools were used to obtain the results presented within these studies, we have been unable to compare them to those presented in the current study. For this same reason, we have also been unable to use them in the construction of the pool of items presented and validated here.

The BPAPS possesses sufficient validity and reliability for measuring barriers to physical activity in low-risk pregnant women who are able to safely engage in physical activity during pregnancy. The engagement and active involvement of pregnant women in this study proved key in evaluating the psychometric properties of the scale. Most of the participants were highly educated (60.33%), and from a self-reported “fairly favorable” socioeconomic status (50%) living in Ilam, which has a particular cultural and geographical context. Future research could usefully be conducted in alternate geographical locations among populations with broader socioeconomic status’ and educational levels. Should future research reveal that the further understanding of barriers to physical activities has an effect upon behavior, the BPAPS may also be useful to midwives in the clinical evaluation of physical activity during pregnancy and the impact of any interventions designed to increase it.

Limitations of this study include a lack of qualitative interviews to provide context to the barriers faced by Iranian women. There is also a lack of generalizability to the instrument, as the items were adapted from studies conducted outside of Iran. Additionally, the scale was used only for assessment of women considered to be experiencing a clinically low risk pregnancy in a single setting. Moreover, only the translated version of the scale from Persian to English was employed here. Thus, future studies could usefully address such gaps in research to further explore the applicability of this tool in other contexts.

## Conclusion

In conclusion, the BPAPS is a valid and reliable tool that can be used to assess the barriers to physical activity among Iranian pregnant women. The BPAPS consists of 4 factors that encompass pregnancy-related intrapersonal barriers, non-pregnancy related intrapersonal barriers, inter-personal barriers, and environmental barriers to provide a holistic assessment of the barriers to physical activity. It would be advantageous for future research to evaluate the usefulness of the BPAPS in other contexts.

## Supplementary Information

**Additional file 1: Supplementary Table S1**. The items were extracted from different studies**Supplementary Table S2**. The total items (48 items) were obtained after face validity**. Supplementary Table S3**. The total items (*n* = 46) were obtained after content validity**. Supplementary Table S4**. The total items were obtained after face and content validity before doing construct validity**. Supplementary Table S5**. Persian version of the ‘Barriers to Physical Activity during Pregnancy Scale’ (BPAPS)**. Supplementary Table S6**. English version of the ‘Barriers to Physical Activity during Pregnancy Scale’ (BPAPS).

## Data Availability

The datasets used and/or analysed during the current study are available from the corresponding author on reasonable request.

## Abbreviations

BPAPS: Barriers to Pregnancy Physical Activity scale
CVI: Content validity index
CVR: Content validity ratio
EBBS: Exercise Benefits/Barriers Scale
ICC: Intraclass Correlation Coefficient
I-CVI: item-level CVI
KMO: Kaiser-Meyer-Olkin
PA: Physical Activity
S-CVI: scale-level CVI
