# Supplementary material for: Development and psychometric testing of the ‘barriers to physical activity during pregnancy scale’ (BPAPS)
Source: BMC Public Health. 2021 Jul 29;21:1483. doi: 10.1186/s12889-021-11511-3 (PMC8323252; doi:10.1186/s12889-021-11511-3)
Supplement: Supplementary file 1 — Additional file 1: Supplementary Table S1. The items were extracted from different studiesSupplementary Table S2. The total items (48 items) were obtained after face validity. Supplementary Table S3. The total items (n = 46) were obtained after content validity. Supplementary Table S4. The total items were obtained after face and content validity before doing construct validity. Supplementary Table S5. Persian version of the ‘Barriers to Physical Activity during Pregnancy Scale’ (BPAPS). Supplementary Table S6. English version of the ‘Barriers to Physical Activity during Pregnancy Scale’ (BPAPS). [file 12889_2021_11511_MOESM1_ESM.docx]

**Supplementary table S1: Items extracted from various studies**

| **No.** | **Items** |
| --- | --- |
| 1 | 1. Physical activity tires me; 2. I am fatigued by physical activity; 3. Physical activity is too hard work for me; 4. Programs where I can be active do not have hours that work for me; 5. There are too few places for me to be active; 6. Family support for exercise; 7. Self-efficacy for exercise; 8. Less family support for exercise and lower self-efficacy for exercise. |
| 2 | 1. Lacking time; 2. Belief that physical activity was dangerous for the mother and unborn baby; 3. Being tired; 4. Being in pain; 5. Embarrassment about appearance; 6. Lack of knowledge about exercising safely; 7. Weather; 8. Lack of social support from partner and family hindered their physical activity behavior; 9. Social support from family and the community; 10. Community safety; 11. Transportation; 12. Education about the safety of exercise and childcare facilitated physical activity behavior. |
| 3 | **Intrapersonal**: 1. Body changes and discomfort; 2. Feeling tired and/or sick; 3. Perceived lack of time; 4. Lack of motivation; 5. Misperceptions; 6. Feeling restricted. **Interpersonal**: 1. Lack of role modeling; 2. Social norms; 3. Lack of emotional support; 4. Lack of informational support. **Environmental**: 1. Weather; 2. Access. |
| 4 | 1. Lack of time, work or social; 2. Conflicts, too busy; 3. Lack of energy, tired, sleepy; 4. Activity causes discomfort/pain or aggravate other medical conditions; 5. Concerned for the baby, does not want to overdo it; 6. No reason; 7. Procrastination; 8. Afraid of injury; 9. Weather—too hot or cold; 10. Caregiving duties; 11. Too costly; 12. Has contractions with activity; 13. Lack of transportation; 14. Others do not encourage activity; 15. Not enough recreational facilities; 16. No one to exercise with; 17. Other; 18. Need for more sleep; 19. Having less time in the day due to sleeping longer; 20. Might harm the baby or bring on premature labor; 21. Want to avoid birthing early; 22. Advice from their physician to slow down; 23. Influence of first pregnancy on activity; 24. Shortness of breath; 25. Musculoskeletal problems associated with physical activity, such as back pain or soreness, as a reason to avoid physical activity; 26. Early contractions; 27. Lack of motivation. |
| 5 | **Intrapersonal Not health related:** 1. Lack of time; 2. Work or social conflicts; 3. Being too busy; 4. Concern about the baby; 5. Not wanting to overdo it; 6. Medical necessity; 7. Dislike of exercise; 8. Lack of motivation. **Intrapersonal Health-related:** 1. Incontinence; 2. Lower back pain; 3. Pelvic pain; 4. Other medical conditions. **Interpersonal:** 1. Having no one to exercise with; 2. Not having access to enough recreational facilities and not being able to afford such facilities. **Policy:** 1. Too costly; 2. Pre-pregnancy PA levels. |
| 6 | 1. Lack of time; 2. Child care responsibilities; 3. Employment obligations; 4. Lack of motivation; 5. Low self-efficacy; 6. Low social support; 7. Perceived lack of self-discipline; 8. Lack of time; 9. A sense of social isolation; 10. Symptoms of pregnancy (fatigue as a major barrier, nausea, and pain); 11. Family and parenting responsibilities and activities; 12. Lack of personal motivation (personal weakness, low self-efficacy, or low self-esteem); 13. Time and employment demands (“too busy” with “time constraints”); 14. Daily life activities provide sufficient exercise; 15. Fear of harm or injury (“nervous” or “fearful “miscarriage” “passing out from exhaustion,” or cause “problem with the pregnancy"); 16. No history or habit of activity or exercise (no previous habit or experience with planned regular physical activity or exercise. |
| 7 | 1. Too tired; 2. Having a lack of time to exercise; 3. Experiencing physical limitations; 4. Joint pain; 5. Swelling; 6. Leg cramps; 7. Sore back; 8. Physical discomfort; 9. Becoming “too big"; 10. Tired (e.g., fatigue, no energy, tiredness); 11. Lack of time (e.g., too busy, other commitments); 12. Physical limitations (e.g., back pain, swelling feet); 13. Work (e.g., long work day, busy at work); 14. Weather (e.g., cold outside, too hot); 15. Lack of motivation (e.g., feeling unmotivated, no desire); 16. Lack of support (e.g., no facility, no classes); 17. Injury/contraindication (e.g., on bed rest, physician’s orders); 18. Childcare duties (e.g., no babysitter, parenting responsibilities, childcare demands). |
| 8 | 1. Physical limitations and restrictions (e.g., nausea, vomiting); 2. Tiredness and fatigue/no energy; 3. Time limits (lacking time); 4. Gaining weight (too big); 5. Caring for other children; 6. Fear of harming self/baby; 7. Bad weather; 8. No motivation/feeling lazy. |
| 9 | Tiredness; 2. Concern with pregnancy complications; 3. Lack of time; 4. Low motivation; 5. Lack of social support; 6. Conflicting information; 7. Neighborhood/environmental issues (e.g., weather); 8. Social isolation; 9. Fear of safety; 10. Lack of transportation; 11. Physical limitation/restrictions; 12. Lack of energy and motivation; 13. Lack of resources; 14. Lack of time; 15. Lack of information; 16. Weather; 17. Physical factors; 18. Social support; 19. Neighborhood safety; 20. Absence/inconvenience of childcare; 21. Dislike of exercise; 22.Perceptions of already being active enough. |
| 10 | 1. Exercising takes too much of my time; 2. Physical activity practice causes fatigue; 3. The places for physical activity practice are too far away; 4. I feel embarrassed to practice physical activity; 5. Physical activity practice is costly; 6. I do not have a convenient schedule for physical activity practice; 7. Practising physical activity tires me; 8. My spouse (or significant other) does not encourage physical activity practice; 9. Physical activity practice takes too much time away from family relationships; 10. I think clothes for physical activity practice show too much of the body; 11. My family members do not encourage me to practice physical activity; 12. Physical activity takes too much time from family responsibilities; 13. Physical activity practice is a difficult task; 14. There are few places for physical activity practice |
| 11 | 1. Nausea; 2. Fatigue; 3. Headache; 4. Back and pelvic pain; 5. Physical limitation such as a growing abdomen; 6. Contractions; 7. Anemia; 8. The fear of harming oneself or the fetus by doing extreme-intensity exercise or the wrong type of exercises; 9. Uncertainty or lack of knowledge about appropriate type of exercise during pregnancy; 10. Sense of exclusion at the fitness center; 11. The pregnancy itself provided an easy excuse. |
| 12 | 1. Pregnancy complaints; 2. Insufficient time; 3. Too much effort to get started; 4. Difficulties due to children and childcare; 5. Sufficient exercise at work/home; 6. Advice from health professional to avoid; 7. Fear of harm to the baby; 8. Difficulty combining with work/studies; 9. Lack of interest; 10. Disease/handicap; 11. Lack of exercise companion; 12. Negative experience with exercise; 13. Lack of availability of exercise options; 14. No experience/never exercise; 15. Social modeling; 16. Anxiety regarding the fetus**.** |
| 13 | 1. Inaccessibility of facilities; 2. Lack of spouse or family support; 3. Places to exercise are too far away; 4. Exercise is too embarrassing; 5. People in exercise clothes look ‘funny’; 6. Places to exercise are too few in number; 7. Exercise is too hard work; 8. Lack of encouragement from spouse and lack of encouragement from family; 9. Costs too much to exercise; 10. Inconvenient facility schedule; 11. Too much time from family relationships; 12. Too much time from family responsibilities; 13. Takes too much of my time; 14. Exercise is tiring; 15. Exercise is fatiguing. |
| 14 | 1. Lower extremities swelling; 2. Extreme weight gain or loss; 3. Back pain; 4. Contraindications to exercise during pregnancy; 5. Lack of feeling to exercise; 6. Insufficient information on exercise; 7. I feel tired to exercise; 8. I do not feel like exercising; 9. I have busy schedule; 10. I have a lot of child care activities; 11. I am afraid of exercise; 12. I do not have sufficient information on exercise. |
| 15 | **Intrapersonal (Pregnancy–related symptoms and limitations):** 1. Fatigue; 2. Tiredness; 3. Lack of energy; 4. Feeling unwell or uncomfortable; 5. Nausea; 6. Back and pelvic pain; 7. Swelling; 8. Soreness; 9. Shortness of breath; 10. Leg cramps; 11. Morning sickness; 12. Contractions; 13. Headache; 14. Anemia; 15. Disease; 16. Bodily changes; 17. The growing body; 18. Physical limitations; 19. No time; 20. Being too busy duo to work; 21. Childcare and family responsibilities; 22. Daily life activities provide sufficient exercise; 23. Lower self-efficacy or discipline; 24. Pregnancy is a time to rest; 25. Dislike of exercise; 26. No habit of exercising; 27. No pre-pregnancy physical activity routine; 28. Problems with body-image; 29. Embarrassment about appearance; 30. Fear of harm the baby or self; 31. Concern with pregnancy complications such as miscarriages and premature labor. **Interpersonal:** 1. Lack of knowledge about how to exercise safely during pregnancy; 2. Lack of health care provider guidance or counseling; 3. Lack access to consistent information; 4. Advice and support on the benefits of physical activity during pregnancy; 5. Insufficient and contradictory information; 6. Lack of accessible information; 7. No one to exercise with; 8. Advice to avoid exercise; 9. No support from family and friends; 10. Conflicting advice from others; 11. Sense of exclusion at the fitness center; 12. Lack of social norms that encourage physical activity; 13. Weather too cold; 14. Weather too hot. **Organizational and policy**: 1. Lack of resources; 2. Unsafe neighborhood; 3. Lack of transportation; 4. Lack of recreational facilities; 5. Too costly; 6. Lack of specific programs for pregnant women. |

**Supplementary table S2. The total items (48 items) were obtained after face validity**

| **No.** | **Items** | **Impact score** |
| --- | --- | --- |
| 1 | Because of family and child-rearing responsibilities and activities, I do not have enough time to do physical activity. | 4.05 |
| 2 | I am active enough in my daily activities. | 1.9 |
| 3 | I do not do any physical activity due to lack of trust in my physical ability. | 4.7 |
| 4 | I cannot be physically active because I do not have a regular schedule for my life. | 4.05 |
| **5** | **Exercise boosts my self-confidence** | **0.58** |
| 6 | Pregnancy is a time for rest. | 4.23 |
| **7** | **I do not enjoy exercising.** | **0.58** |
| 8 | I cannot be physically active, because I do not have a physical activity habit. | 3.36 |
| 9 | I can’t exercise because I do not have exercise experience. | 4.9 |
| 10 | I cannot exercise because of my negative experiences with exercise. | 3.52 |
| 11 | I do not have the patience to exercise. | 2.73 |
| 12 | Exercise makes me tired. | 4.5 |
| 13 | Physical activity is too hard work for me. | 4.23 |
| 14 | I cannot exercise because of my mental image of the changes in my body. | 3.28 |
| 15 | I cannot be physically active due to lethargy and lack of energy. | 3.2 |
| 16 | Because of sleepiness, I can’t be physically active. | 3.87 |
| 17 | I cannot be physically active because of back pain and hip pain. | 1.75 |
| 18 | The heavy feeling (swelling and weight) makes me unable to be physically active. | 4.32 |
| 19 | I cannot be physically involved because of shortness of breath. | 2.94 |
| 20 | I cannot be physically active due to pregnancy gastrointestinal problems (such as nausea, vomiting, and heartburn). | 2.1 |
| 21 | I cannot be physically active because of headache. | 2.73 |
| 22 | I cannot be physically active because of anemia. | 3.52 |
| 23 | I cannot be physically active because of my abdominal size and appearance. | 4.14 |
| 24 | I do not exercise because I am afraid of hurting myself. | 3.78 |
| 25 | I do not exercise because I am afraid of hurting my unborn child. | 3.78 |
| 26 | I am concerned about the side effects on pregnancy, such as miscarriage and premature labor. | 1.58 |
| 27 | I cannot exercise due to the lack of a companion. | 2.59 |
| 28 | My wife and family do not support me in exercising during pregnancy. | 2.59 |
| 29 | My friends and relatives forbid me from doing physical activity during pregnancy. | 4.4 |
| 30 | I feel ashamed of exercising in front of others. | 4.5 |
| 31 | In our culture, physical exercise is not common among pregnant women. | 4.9 |
| 32 | Caregivers and health workers do not recommend exercising during pregnancy. | 4.4 |
| 33 | The doctor or midwife does not offer advice on how to perform physical activity and how to be safe during pregnancy. | 1.65 |
| 34 | Staff and health care providers do not provide guidance or advice on how to exercise during pregnancy. | 2.59 |
| 35 | I do not do physical activity because I do not have access to the full knowledge and awareness of physical activity during pregnancy. | 1.75 |
| 36 | The doctor or midwife does not offer advice on the benefits of physical activity during pregnancy. | 1.58 |
| 37 | Lack of indoor space keeps me from exercising. | 4.4 |
| 38 | Air pollution keeps me from doing physical activity outdoors. | 4.5 |
| 39 | It is hard for me to do physical activity in unfavorable weather (too cold/hot). | 3.28 |
| 40 | I cannot afford to do physical activity. | 3.2 |
| 41 | There is a great distance from my home to physical activity facilities. | 3.87 |
| 42 | There are very few places where I can do physical activity. | 1.75 |
| 43 | I do not do physical activity because I do not have access to the proper means of transport. | 3.28 |
| 44 | Physical activity programs for pregnant women are not available. | 3.2 |
| 45 | Parks are dangerous and unsuitable for pregnant women to perform physical activity. | 3.87 |
| 46 | I do not exercise because of a lack of space at home. | 1.75 |
| 47 | I find physical exercise boring. | 3.2 |
| 48 | I do not have happy memories of exercising. | 1.58 |

**Supplementary table S3. The total items (n = 46) were obtained after content validity**

| **No.** | **Items** | CVI | CVR |
| --- | --- | --- | --- |
| 1 | Because of family and child-rearing responsibilities and activities, I do not have enough time to do physical activity. | 0.9 | 0.8 |
| 2 | I am active enough in my daily activities. | 0.8 | 1 |
| 3 | I do not do any physical activity due to lack of trust in my physical ability. | 0.7 | 1 |
| 4 | I cannot be physically active because I do not have a regular schedule for my life. | 1 | 1 |
| 5 | Pregnancy is a time for rest. | 0.9 | 0.8 |
| 6 | I cannot be physically active because I do not have a physical activity habit. | 0.8 | 0.8 |
| 7 | I can’t exercise because I do not have exercise experience. | 0.7 | 1 |
| 8 | **I cannot exercise because of my negative experiences with exercise.** | **0.4** | - |
| 9 | I do not have the patience to exercise. | 0.9 | 0.8 |
| 10 | Exercise makes me tired. | 0.8 | 1 |
| 11 | Physical activity is too hard work for me. | 0.7 | 1 |
| 12 | **I cannot exercise because of my mental image of the changes in my body.** | 1 | **0.2** |
| 13 | I cannot be physically active due to lethargy and lack of energy. | 0.9 | 1 |
| 14 | Because of sleepiness, I cannot be physically active. | 0.8 | 1 |
| 15 | I cannot be physically active because of back pain and hip pain. | 0.7 | 0.8 |
| 16 | The heavy feeling (swelling and weight) makes me unable to be physically active. | 1 | 1 |
| 17 | I cannot be physically involved because of shortness of breath. | 0.9 | 1 |
| 18 | I cannot be physically active due to pregnancy gastrointestinal problems (such as nausea, vomiting, and heartburn). | 0.8 | 1 |
| 19 | **I cannot be physically active because of headache.** | 0.7 | **0.6** |
| 20 | **I cannot be physically active because of anemia.** | 1 | **0.4** |
| 21 | I cannot be physically active because of my abdominal size and appearance. | 0.9 | 1 |
| 22 | I do not exercise because I am afraid of hurting myself. | 0.8 | 1 |
| 23 | I do not exercise because I am afraid of hurting my unborn child. | 0.7 | 0.8 |
| 24 | I am concerned about the side effects on pregnancy, such as miscarriage and premature labor. | 1 | 1 |
| 25 | **I cannot exercise due to the lack of a companion.** | **0.2** | - |
| 26 | My wife and family do not support me in exercising during pregnancy. | 0.9 | 0.8 |
| 27 | My friends and relatives forbid me from doing physical activity during pregnancy. | 0.8 | 1 |
| 28 | I feel ashamed of exercising in front of others. | 0.7 | 1 |
| 29 | In our culture, physical exercise is not common among pregnant women. | 1 | 0.8 |
| 30 | **Caregivers and health workers do not recommend exercising during pregnancy.** | 0.9 | **0.4** |
| 31 | The doctor or midwife does not offer advice on how to perform physical activity and how to be safe during pregnancy. | 0.8 | 1 |
| 32 | **Staff and health care providers do not provide guidance or advice on how to exercise during pregnancy.** | 0.7 | **0** |
| 33 | I do not do physical activity because I do not have access to the full knowledge and awareness of physical activity during pregnancy. | 1 | 1 |
| 34 | The doctor or midwife does not offer advice on the benefits of physical activity during pregnancy. | 0.9 | 1 |
| 35 | **Lack of indoor space keeps me from exercising.** | 0.8 | **0.2** |
| 36 | Air pollution keeps me from doing physical activity outdoors. | 0.7 | 1 |
| 37 | It is hard for me to do physical activity in unfavorable weather (too cold/hot). | 1 | 1 |
| 38 | I cannot afford to do physical activity. | 0.9 | 0.8 |
| 39 | There is a great distance from my home to physical activity facilities. | 0.8 | 1 |
| 40 | There are very few places where I can do physical activity. | 0.7 | 1 |
| 41 | I do not do physical activity because I do not have access to the proper means of transport. | 1 | 0.8 |
| 42 | Physical activity programs for pregnant women are not available. | 0.9 | 1 |
| 43 | Parks are dangerous and unsuitable for pregnant women to perform physical activity. | 0.8 | 1 |
| 44 | I do not exercise because of a lack of space at home. | 0.7 | 0.8 |
| 45 | I find physical exercise boring. | 1 | 1 |
| 46 | I do not have happy memories of exercising. | 0.9 | 1 |

**Supplementary table S4. The total items were obtained after face and content validity before doing construct validity**

| **No.** | **Items** |
| --- | --- |
| 1 | Because of sleepiness, I cannot be physically active. |
| 2 | I cannot be physically active due to lethargy and lack of energy. |
| 3 | I cannot be physically active because I do not have a physical activity habit. |
| 4 | Pregnancy is a time for rest. |
| 5 | I cannot exercise because I do not have exercise experience. |
| 6 | The heavy feeling (swelling and weight) makes me unable to be physically active. |
| 7 | I cannot be physically active because of my abdominal size and appearance. |
| 8 | I cannot be physically active because of pain (such as back pain, hip pain, headache, etc.). |
| 9 | I cannot be physically involved because of shortness of breath. |
| 10 | I am concerned about the side effects on pregnancy, such as miscarriage and premature labor. |
| 11 | I cannot be physically active due to pregnancy gastrointestinal problems (such as nausea, vomiting, and heartburn). |
| 12 | I do not have happy memories of exercising. |
| 13 | I do not have the patience to exercise. |
| 14 | Physical activity is too hard work for me. |
| 15 | I do not do any physical activity due to lack of trust in my physical ability. |
| 16 | I find physical exercise boring. |
| 17 | Exercise makes me tired. |
| 18 | I cannot be physically active because I do not have a regular schedule for my life. |
| 19 | Because of family and child-rearing responsibilities and activities, I do not have enough time to do physical activity. |
| 20 | I feel ashamed of exercising in front of others. |
| 21 | In our culture, physical exercise is not common among pregnant women. |
| 22 | I am active enough in my daily activities. |
| 23 | I do not do physical activity because I do not have access to the full knowledge and awareness of physical activity during pregnancy. |
| 24 | My friends and relatives forbid me from doing physical activity during pregnancy. |
| 25 | The doctor or midwife does not offer advice on the benefits of physical activity during pregnancy. |
| 26 | My wife and family do not support me in exercising during pregnancy. |
| 27 | The doctor or midwife does not offer advice on how to perform physical activity and how to be safe during pregnancy. |
| 28 | Air pollution keeps me from doing physical activity outdoors. |
| 29 | I do not do physical activity because I do not have access to the proper means of transport. |
| 30 | I do not exercise because I am afraid of hurting myself. |
| 31 | I do not exercise because I am afraid of hurting my unborn child. |
| 32 | It is hard for me to do physical activity in unfavorable weather (too cold/hot). |
| 33 | I cannot afford to do physical activity. |
| 34 | Physical activity programs for pregnant women are not available. |
| 35 | Parks are dangerous and unsuitable for pregnant women to perform physical activity in. |
| 36 | I do not exercise because of a lack of space at home. |
| 37 | There is a great distance from my home to physical activity facilities. |
| 38 | There are very few places where I can do physical activity. |

**Supplementary Table S5. Persian version of the ‘Barriers to Physical Activity during Pregnancy Scale’ (BPAPS)**

نسخه فارسی مقیاس موانع فعالیت فیزیکی در بارداری

| **خیلی مخالف** | **مخالف** | **بدون نظر** | **موافق** | **خیلی موافق** | **عبارات** |
| --- | --- | --- | --- | --- | --- |
|  |  |  |  |  | فعالیت فیزیکی برای من کار سختی است. |
|  |  |  |  |  | همیشه به دلیل خواب آلودگی نمی­توانم فعالیت فیزیکی داشته باشم. |
|  |  |  |  |  | به دلیل بی­حالی و کمبود انرژی نمی­توانم فعالیت فیزیکی داشته باشم. |
|  |  |  |  |  | به دلیل اعتماد نداشتن به توانایی­ بدنی ام نمی­توانم فعالیت فیزیکی داشته باشم. |
|  |  |  |  |  | حوصله انجام فعالیت فیزیکی ندارم. |
|  |  |  |  |  | به خاطر اینکه عادت به فعالیت فیزیکی ندارم نمی­توانم آن را انجام دهم. |
|  |  |  |  |  | بارداری زمانی برای استراحت کردن است. |
|  |  |  |  |  | به دلیل نداشتن برنامه منظم در زندگی، نمی­توانم فعالیت فیزیکی داشته باشم. |
|  |  |  |  |  | در جامعه ی ما داشتن فعالیت فیزیکی در زنان باردار رسم نیست. |
|  |  |  |  |  | به دلیل مراقبت از فرزندانم و مسئولیت­های متعدد در خانواده، وقت کافی برای فعالیت فیزیکی ندارم. |
|  |  |  |  |  | به دلیل احساس سنگینی ( ورم و اضافه وزن) نمی­توانم فعالیت فیزیکی داشته باشم. |
|  |  |  |  |  | به دلیل بزرگ شدن شکم و تغییرات ظاهری بدنم نمی­توانم فعالیت فیزیکی داشته باشم. |
|  |  |  |  |  | به دلیل داشتن درد (مانند درد پشت، درد لگن و سر درد) نمی­توانم فعالیت فیزیکی داشته باشم. |
|  |  |  |  |  | به دلیل احساس تنگی نفس نمی­توانم فعالیت فیزیکی داشته باشم. |
|  |  |  |  |  | فکر می­کنم فعالیت فیزیکی موجب سقط و زایمان زودرس در زنان باردار می­شود. |
|  |  |  |  |  | به دلیل داشتن مشکلات گوارشی (مانند تهوع و استفراغ صبحگاهی، سوزش سر دل) نمی­توانم فعالیت فیزیکی داشته باشم. |
|  |  |  |  |  | آلودگی هوا مانع می­شود که در هوای آزاد فعالیت فیزیکی داشته باشم. |
|  |  |  |  |  | چون به وسیله نقلیه مناسب برای رفت و آمد به مکان­های ورزشی دسترسی ندارم، نمی­توانم فعالیت فیزیکی کافی داشته باشم/ |
|  |  |  |  |  | برای من فعالیت فیزیکی در آب و هوای نامطلوب (سرد و گرم) مشکل است. |
|  |  |  |  |  | به دلیل عدم دسترسی به اطلاعات کامل در زمینه فعالیت فیزیکی در دوران بارداری، نمی­توانم فعالیت فیزیکی کافی داشته باشم. |
|  |  |  |  |  | دوستان و اطرافیانم مرا از انجام فعالیت فیزیکی در بارداری منع می­کنند. |
|  |  |  |  |  | قادر نیستم هزینه­های مربوط به شرکت در برنامه­های فعالیت­ فیزیکی را پرداخت نمایم. |
|  |  |  |  |  | پزشک/ ماما در خصوص مزایای فعالیت فیزیکی در دوران بارداری راهنمایی و مشاوره ارائه نمی­کنند. |
|  |  |  |  |  | پزشک/ ماما در خصوص نحوه انجام و ایمن بودن فعالیت فیزیکی در بارداری راهنمایی و مشاوره ارائه نمی­کنند. |
|  |  |  |  |  | برنامه­های فعالیت فیزیکی مخصوص زنان باردار وجود ندارد. |
|  |  |  |  |  | پارک­ها برای فعالیت فیزیکی زنان باردار ناامن و نامناسب است. |
|  |  |  |  |  | به دلیل نداشتن فضای مناسب در منزل، فعالیت فیزیکی نمی­توانم داشته باشم. |
|  |  |  |  |  | فاصله مکان­های انجام فعالیت فیزیکی از منزل من بسیار زیاد است. |
|  |  |  |  |  | مکان­های بسیار کمی برای فعالیت فیزیکی من وجود دارد. |

**Supplementary Table S6. English version of the ‘Barriers to Physical Activity during Pregnancy Scale’ (BPAPS)**

| **Items** | **Strongly agree** | **Agree** | **Neutral** | **Disagree** | **Strongly disagree** |
| --- | --- | --- | --- | --- | --- |
| Physical activity is too hard work for me. |  |  |  |  |  |
| I cannot be physically active because of drowsiness. |  |  |  |  |  |
| I cannot be physically active because of lethargy/lack of energy. |  |  |  |  |  |
| I do not do physical activity because of a lack of confidence in my physical ability. |  |  |  |  |  |
| I do not have the patience to do physical activity. |  |  |  |  |  |
| I cannot be physically active because I do not have physical activity habits. |  |  |  |  |  |
| Pregnancy is a time to rest. |  |  |  |  |  |
| I cannot be physically active because I do not have a regular schedule in life. |  |  |  |  |  |
| In our society, it is not customary for pregnant women to do physical activity. |  |  |  |  |  |
| Because of family and childrearing responsibilities/activities I do not have enough time to do physical activity. |  |  |  |  |  |
| I cannot be physically active because of the heavy feeling of pregnancy (swelling and/or weight). |  |  |  |  |  |
| I cannot be physically active because of my abdominal size and appearance. |  |  |  |  |  |
| I cannot be physically active because of pain (such as back pain, hip pain, and/or headache). |  |  |  |  |  |
| I cannot be physically active because of shortness of breath. |  |  |  |  |  |
| I am concerned by possible pregnancy complications such as miscarriages and premature labor. |  |  |  |  |  |
| I cannot be physically active because of pregnancy gastrointestinal problems (such as nausea, vomiting, and heart burn). |  |  |  |  |  |
| Air pollution prevents me from doing physical activity outdoors. |  |  |  |  |  |
| I do not do physical activity because I do not have access to a suitable vehicle for transportation. |  |  |  |  |  |
| It is difficult for me to do physical activity in unfavorable weather (too cold/hot). |  |  |  |  |  |
| I do not do physical activity because I do not have access to complete information about physical activity during pregnancy. |  |  |  |  |  |
| My friends and relatives forbid me from doing physical activity during pregnancy. |  |  |  |  |  |
| I am not able to pay for physical activities. |  |  |  |  |  |
| The physician/midwife does not provide advice on the benefits of physical activity during pregnancy. |  |  |  |  |  |
| The physician/midwife does not provide advice on how to do physical activity safely during pregnancy. |  |  |  |  |  |
| There are no specific physical activity programs designed for pregnant women. |  |  |  |  |  |
| Parks are unsafe and unsuitable for pregnant women to do physical activity. |  |  |  |  |  |
| I do not do physical activity because of a lack of space at home. |  |  |  |  |  |
| There is too great a distance from my home to facilities designed for physical activity. |  |  |  |  |  |
| There are very few places for me to do physical activity. |  |  |  |  |  |
